# The force awakens: metastatic dormant cancer cells

**DOI:** 10.1038/s12276-020-0423-z

**Published:** 2020-04-16

**Authors:** So-Yeon Park, Jeong-Seok Nam

**Affiliations:** 1 School of Life Sciences, Gwangju Institute of Science and Technology, Gwangju, 61005 Republic of Korea; 2 Cell Logistics Research Center, Gwangju Institute of Science and Technology, Gwangju, 61005 Republic of Korea

**Keywords:** Cancer microenvironment, Metastasis

## Abstract

Recurrent cancer that spreads to distant sites is the leading cause of disease-related death among cancer patients. Cancer cells are likely to disseminate during cancer progression, and some may enter dormancy, remaining viable but not increasing. These dormant cancer cells (DCCs) are rarely detectable with current diagnostic systems. Moreover, they can interpret homoeostatic signals from the microenvironment, thereby evading immune surveillance and chemotherapy. Eventually, DCCs can reawaken in response to signals, which are not yet fully understood, resulting in recurrence and metastasis. Therefore, understanding the biology of DCC reawakening is key to preventing metastasis. Over the last decade, a growing body of literature has revealed the mechanisms involved in cancer dormancy and reawakening. The cytotoxic activity of immune cells can cause cancer cells to enter a dormant state, and chronic inflammation can reactivate cancer proliferation at distant sites. Upon the binding of circulating DCCs to extracellular molecules, various signaling cascades are activated and reinitiate cell proliferation. In the present review, we attempt to consolidate the existing literature to provide a framework for the understanding of this crucial step in cancer progression.

## Cancer spread: How to let sleeping cells lie

Preventing dormant cancer cells (DCCs) from reawakening could be key to preventing cancer recurrence. During cancer progression, dormant tumor cells can travel through the bloodstream, reawakening to form tumors in distant tissues. These tumors, known as metastases, are difficult to treat. The signals that cue DCCs to enter and exit dormancy are poorly understood. In a review, Jeong-Seok Nam and So-Yeon Park at the Gwangju Institute of Science and Technology in South Korea report that DCCs often enter dormancy to evade attack by immune cells. Once in a new location, DCCs receive signals from the surrounding tissue, which can trigger tumor development. DCCs can also be reactivated by chronic inflammation. Signals that block reactivation of DCCs are currently being tested as potential therapeutics, and may help in the fight against this leading cause of death from cancer.

## Introduction

The primary treatment for cancer is the surgical removal of cancer cells, which is often combined with chemoradiotherapy to kill surgically inaccessible cancer cells throughout the body. However, even patients who are considered clinically free of cancer cells after initial treatment frequently relapse with distant metastasis. Such metastatic outgrowth rapidly becomes uncontrollable with chemoradiation and manages to seed additional metastatic colonies, resulting in the disruption of vital organ function. Although the clinical importance of metastasis is therefore apparent, its underlying mechanisms remain unclear.

Metastasis is considered a series of linear events, termed the invasion–metastasis cascade^1^. The initiation step of metastasis begins when cancer cells at the primary tumor growth site foster basement membrane degradation and enter the underlying interstitial matrix^2^. During this process, cancer cells usually promote vascularization in tumor tissues to sculpt a permissive microenvironment for cancer cell proliferation and gain access to the bloodstream^3^. Once cancer cells successfully penetrate into the blood or lymphatic circulatory system, they can disseminate throughout the body. In circulation, cancer cells are likely to exhibit mitotic arrest through reversible G0-G1 arrest, termed quiescence, in which they remain viable but do not increase. These dormant cancer cells (DCCs) are more susceptible to antiproliferative drugs. More recently, these circulating DCCs have been shown to evade immune surveillance by expressing programmed death ligand 1 (PDL-1); thus, they can persist for an extended period^4,5^. At some point, DCCs reach distant organs and infiltrate into the stroma, although they cannot grow into macroscopic lesions until they escape dormancy. This period is termed “metastatic cancer dormancy” and occurs between initial therapy and metastatic relapse. Eventually, in response to microenvironmental cues, DCCs gain the ability to re-enter the cell cycle and adapt to their new microenvironment, thereby progressing to metastatic outgrowth. Therefore, understanding the biology of DCC reawakening is key to preventing metastasis.

A growing body of research has provided insight into the molecular mechanisms of cellular dormancy and reactivation. Central to these mechanisms is crosstalk between cancer cells and their microenvironment, which is affected by complex interactions between cancer cells and stromal cells and surrounding extracellular matrix (ECM) components, as well as host immunity. After a long period in the bloodstream, DCCs eventually reach distant organs and encounter a new composition of ECM produced from the local stromal cells. Then, the binding of membrane receptors on DCCs activates various signaling cascades, driving cell cycle promotion and breaking dormancy. Meanwhile, the host immune system initially acts as a tumor suppressor but eventually favors cancer progression and promotes metastatic outgrowth by reactivating DCCs. In the present review, we focused on these cellular and acellular factors that reawaken DCCs and contribute to metastasis.

## Primary molecular mechanisms underlying cancer cell dormancy

An overwhelming amount of evidence supports the notion that extracellular signal-regulated kinase (ERK) activation has a determinant role in whether cancer cells will proliferate or enter a state of dormancy. Persistently proliferating cancer cells exhibit constitutive ERK activation, which permits Go-G1-S phase transition and cell division^6,7^. During ERK-induced proliferation, a high level of p38 mitogen-activated protein kinase (p38) activity functions as an inhibitory regulator of ERK and prevents cell proliferation by inducing G0-G1 arrest or triggering senescence and apoptosis^8–10^. Indeed, a luciferase reporter system visualized the in vivo ERK and p38 MAPK activities and provided direct evidence of p38/ERK activity as an indicator of DCCs in various types of cancer, including breast cancer, prostate cancer, melanoma, and fibrosarcoma^8^. Cancer cells with p38^low^/ERK^high^ activity were highly proliferative in vivo, whereas those with p38^high^/ERK^low^ activity were incapable of proliferation without increased apoptosis, suggesting that they were dormant in vivo. Meanwhile, multiple pharmacological and genetic interventions that change the balance of p38/ERK activity in favor of ERK were able to break in vivo dormancy and induce cancer growth. Thus, it seems that regulatory factors that can change the signaling balance between ERK and p38 activities have a profound influence on whether cancer cells grow or remain dormant^11^.

Transforming growth factor-β2 (TGF-β2) is secreted from bone marrow-derived cells and thus is relatively abundant. TGF-β2 binds to its receptors, TGF-β receptor-I (TGF-β-RI) and TGF-β-RIII, on cancer cell membranes and induces p38^high^/ERK^low^ signaling^12^. The subsequent activation of Smad1/5 increases the expression of DEC2/SHARP1 and p27 and downregulates cyclin-dependent kinase 4 (CDK4), which collectively facilitates the transition into cellular quiescence^12,13^. The production of TGF-β1/2 is increased during osteoblast differentiation, along with that of bone morphogenetic protein (BMP) family proteins. Both TGF-β1 and BMP-3b induce cancer cell quiescence. TGF-β-RIII participates in both TGF-β1- and BMP-3b-induced dormancy and activates the phosphorylation of retinoblastoma through p38 MAPK activation. On the other hand, in the lung, where stromal TGF-β2 secretion is low, ERK activation is restored, and DCCs transition into a highly proliferative state, fueling multiorgan metastasis^12^. Therefore, upon the exit of DCCs from bone marrow, the lack of growth factors can shift the balance of p38 MAPK and ERK activities toward ERK activation, creating a permissive microenvironment for metastatic outgrowth.

The urokinase plasminogen activator (uPA) system has been implicated in a shift from cancer dormancy to proliferation by mediating EGFR signaling^14^. Numerous types of cells, including epithelial cells, immune cells, and fibroblasts, produce and secrete uPA. uPA binds to its receptor (uPAR) and initiates a proteolytic cascade, resulting in the conversion of plasminogen into plasmin^15^. Plasmin degrades a wide range of extracellular components through its proteolytic activity and activates other enzymatic proteins, such as metalloproteinases, thereby promoting cancer invasion. Independent of catalytic activity, the uPAR–uPA interaction leads to the activation of integrin and epidermal growth factor receptor (EGFR) signaling, which consecutively activates ERK1/2 and lowers p38 activities, promoting mitotic cascades^8,16,17^. However, DCCs have been reported to express a low level of uPAR; thus, they exhibit a low level of integrin and EGFR activation, resulting in a p38^high^/ERK^low^ activity ratio^8^. Additionally, p38^high^/ERK^low^ facilitates G0-G1 arrest by regulating a variety of transcription factors (TFs), such as nuclear receptor subfamily 2 group F member 1 (NR2F1), basic helix-loop-helix protein 3 (BHLHB3 or DEC2), and cyclin-dependent kinase inhibitors (p27 and p21), and downregulates G1 exit-promoting TFs, such as FOXM1 and c-Jun^18^. Therefore, this combinatorial regulation of TFs by p38^high^/ERK^low^ activity is responsible for the quiescence program in DCCs^11^.

Additional studies have suggested that high p38 activity is linked to the survival of DCCs and related to endoplasmic reticulum (ER) stress. High p38 activity inhibits Bax activation by increasing the expression of the ER chaperone BiP/Grp78, thereby rendering DCCs highly resistant to chemotherapy^19^. The activating transcription factor 6α (ATF6α), which is translocated from the ER to the nucleus to serve as a TF upon ER stress, is persistently activated in DCCs in a p38-dependent manner^20^. ATF6α transcriptionally induces Rheb, a small GTPase, and transduces survival signals such as mTOR and downstream S6K and S6RP phosphorylation. Knockdown of ATF6α or Rheb by RNA interference was sufficient to induce apoptosis in DCCs and remove DCCs during their quiescent phase^20^. This suggests that high p38 activity causes growth arrest in DCCs and simultaneously may activate the dormancy-specific survival signaling pathways that enable DCCs to resist microenvironmental and genotoxic stress.

Furthermore, some kinds of stroma-derived ligands are known to induce cancer cell dormancy in multiple types of cancer. For example, growth arrest-specific protein 6 (GAS6) has been shown to induce dormancy in several kinds of cancer cells that infiltrate the bone marrow. GAS6 is known to bind to the Tyro3, Axl, and Mer (TAM) family of receptor tyrosine kinases, thereby activating multiple downstream signaling pathways, including mitogen-activated protein kinase (MAPK) and phosphoinositide 3-kinase (PI3K)/Akt pathways^21^. In particular, GAS6 promotes the transition of cancer cells into DCCs in the bone marrow. Mechanistically, osteoblasts secrete GAS6 upon their contact with leukemia cells, and the binding of GAS6 to Mer on the surface of leukemia cells facilitates the entry of leukemia cells into G0/G1 arrest^22^. Similarly, in bone marrow, GAS6 from osteoblasts activates TAM family receptors on prostate cancer cells and switches on dormancy in proliferative cancer cells^23^. Additionally, BMP7, produced from bone stromal cells, can induce dormancy in prostate cancer cells by activating p38 signaling^24^. Mechanistically, binding of BMP7 to its receptor BMP receptor 2 (BMPR2) on prostate cancer cells activates p38 signaling; in turn, it induces reversible growth arrest by increasing the expression of the cell cycle inhibitor p21 and the metastasis suppressor gene NDRG1 (N-myc downstream-regulated gene 1). These data together show that many of these dormancy-inducing cytokines from the stroma can promote the p38^high^/ERK^low^ state in the absence of proliferative signaling, resulting in G0 cell cycle arrest and cancer dormancy.

## Breaking of cellular dormancy by microenvironmental cues

Integrins are transmembranous heterodimeric glycoproteins that mediate cell-to-cell and cell-to-ECM signaling cascades. Integrin signaling activates multiple intermediaries, including cytosolic tyrosine kinases, and is involved in the regulation of cell proliferation, survival, and motility in both cancer and normal healthy cells^25^. Numerous studies have provided evidence that integrin signaling, particularly β-1 integrin, is a critical regulator in the switch from cellular dormancy to metastatic growth in vitro and in vivo^25–29^. Loss of β-1 integrin signaling by downregulation of the uPA-uPAR interaction appears to promote the shift from a proliferative to a dormant state in cancer cells^8^. The inhibition of β-1 integrin signaling by antibody treatment induced the growth arrest of mammary cancer cells in a three-dimensional basement membrane assay^30^. The removal of the anti-β-1 integrin antibody reversed cell cycle arrest and reinitiated cancer cell growth. Focal adhesion kinase (FAK) is a downstream molecule of β-1 integrin and has been implicated in the regulation of cancer cell dormancy. In a mouse mammary tumor virus (MMTV) transgenic breast cancer mouse model, the Cre-LoxP-mediated deletion of β-integrin results in a decrease in FAK phosphorylation, reduced cell proliferation, and growth arrest of tumor burden in vivo^31^. Similarly, the growth ability of a highly metastatic D2A1 mammary carcinoma cell was significantly dependent on the presence of fibronectin, β-1 integrin signaling, and downstream phosphorylation of the myosin complex in three-dimensional cell culture, suggesting that the upregulation of β-1 signaling enabled DCCs to re-enter the cell cycle^26^.

An additional in vivo study revealed that metastatic outgrowth of the mouse mammary cancer cell lines D2.0R and D2A1 was dependent on β1-integrin signaling^32^. Binding of collagen to integrin receptors resulted in FAK/SRC activation and subsequent ERK phosphorylation. Integrin-mediated ERK activation induced cell proliferation, driving metastatic outgrowth. These data suggest that the interaction between β1-integrin/FAK and the MAPK pathway is essential for cancer cell growth. Meanwhile, noncanonical discoidin domain receptor 1 (DDR) signaling is also activated by binding to collagen, and it is known to activate cancer cell proliferation at metastatic sites^33^. Mechanistically, tetraspanin transmembrane 4 L six family member 1 (TM4SF1) couples DDR1 to syntenin 2 and then activates protein kinase C alpha (PKCα). Activated PKCα subsequently phosphorylates Janus kinase 2 (JAK2) to drive noncanonical DDR1 signaling through phosphorylation of signal transducer and activator of transcription 3 (STAT3). In cancer, constitutive activation of STAT3 increases the transcription of cell cycle regulators, such as c-Myc and cyclin D, and promotes cancer cell proliferation. Consistently, histopathologic analysis of metastatic murine breast cancer cells has identified that micrometastatic tissues are surrounded by collagen. In metastatic tissues, the majority of cancer cells apart from collagen are dormant, whereas those nearby collagen are proliferative. These findings indicate how the interaction between DCCs and the ECM microenvironment influences cancer cell behavior and metastatic reactivation.

Furthermore, Wnt signaling has been implicated as a mediator during ECM-induced DCC reactivation. Wnt signaling is known to control diverse biological processes and is a well-known proliferation inducer. Wnt activation promotes G1-to-S progression through both transcriptional and nontranscriptional regulation of cyclin D1, cyclin E1, and c-myc^34^. Therefore, inhibition of Wnt signaling by secretion of Dickkopf WNT signaling pathway inhibitor 1 (DKK1) is one mechanism by which cancer cells enter quiescence^35^. Tenascin C, initially produced by metastasis-initiating cancer cells and later secreted from stromal fibroblasts, is known to support the metastatic outgrowth of breast cancer cells by promoting Wnt signaling. Tenascin C binds to syndecan-4, a coreceptor of the Wnt receptor Frizzled-7, thereby enhancing Wnt signaling activation and facilitating metastatic colonization. Additionally, periostin has the ability to recruit Wnt ligands; thus, it can increase the presentation of Wnt ligands to cancer cells. Periostin is mainly produced from stromal fibroblasts upon TGF-β activation and can be secreted from endothelial tip cells that reside in new vascular sprouts. Thus, periostin is abundant in micrometastatic lesions undergoing neoangiogenesis and is a profound factor for a permissive microenvironment of cancer metastasis. Moreover, both tenascin C and periostin can foster integrin signaling through an indirect pathway; they coassemble with fibronectin and modulate its adhesiveness and stiffness, which collectively increase the integrin signaling capacity.

Collectively, these facts suggest that the ECM components from metastasis-initiating cancer cells and stromal cells may sculpt a permissive niche, facilitating the activation of signaling pathways that support metastatic cell proliferation.

## Chronic inflammation awakens dormant cells

Growing evidence has suggested that chronic inflammation is involved in cancer development. For example, patients with inflammatory bowel disease are at higher risk of colorectal cancer development. Hepatitis and fatty liver disease correlate with the incidence of liver cancer development. Acid reflux esophagitis can cause esophageal cancer. Chronic *Helicobacter* infection is the leading cause of stomach cancer. During inflammation, free radicals such as reactive oxygen and nitrogen species (RONS) increase and induce double-strand breaks in DNA, which are potently mutagenic if not accurately and promptly repaired, thereby facilitating the transformation of normal healthy cells to cancer cells^36^. Moreover, free radicals can trigger a wide range of signaling pathways, including MAPK/ERK, PI3K/Akt, and IκB kinase/nuclear factor kappa-light-chain-enhancer of activated B cells (NFκB), that lead to cancer malignancy^37^. However, not all individuals who have experienced chronic inflammatory diseases eventually develop cancer in their lifetime. In situ carcinoma can be found in the lesion without any chronic inflammation. These phenomena raised the question about whether a cause-effect relationship exists between chronic inflammation and cancer. One of the possible explanations for this conflicting evidence may be that reawakening DCCs could be a key factor for cancer development from chronic inflammation. For instance, chronic inflammation supports angiogenesis, which breaks cancer dormancy by supplying sufficient oxygen and nutrients and facilitates cancer growth^38^. Moreover, there is a strong correlation between inflammation and recurrence of cancer, including recurrence of endometrial^39^, oral^40^, and breast cancer^41,42^. The escape of cancer from dormancy can be induced by the inflammatory cytokine interferon-gamma (IFN-γ)^43–46^. In addition, the correlation between the high levels of serum inflammatory cytokines and cancer recurrence supports this hypothesis. In a cohort consisting of 734 breast cancer patients, high levels of circulating acute-phase proteins (APPs) were positively correlated with distant recurrence^47^. Additionally, C-reactive protein (CRP) and interleukin 6 (IL-6), other serum inflammatory markers, have shown their possibilities as posttreatment prognostic monitoring factors for predicting the risk of cancer recurrence and patient death^48–50^. Hepatocyte CRP secretion is controlled by interleukin 6 (IL-6). The synthesis of CRP is stimulated by interleukin-1 (IL-1) and tumor necrosis factor (TNF). A rise in serum levels of CRP often reflects tissue damage. Collectively, these data support the hypothesis that inflammation can be the DCC reawakening factor and therefore can function as a cancer-promoting factor.

Chronic inflammation can induce epigenetic alterations and DNA mutations in tumor suppressor genes, thereby facilitating carcinogenesis. Fortunately, the immune system can recognize these mutant protein antigens of cancer cells and can attack cancer cells, serving as a critical mechanism of metastatic dormancy, so-called immunogenic cancer dormancy^51,52^. For instance, CD8+ T cells have a cytostatic effect on cancer cells, thereby allowing early disseminated cancer cells to stay in a dormant state at metastatic sites^53^. In some experimental models, removal of CD8+ T cells resulted in outgrowth of DCCs and induced cancer recurrence^53^. However, chronic inflammation can also facilitate other mechanisms that promote the reactivation of DCCs. For instance, studies in a pancreatic cancer mouse model demonstrated that circulating cancer cells underwent epithelial to mesenchymal transition (EMT) and seeded metastatic colonies in the liver. In this process, the rate of EMT and invasive potential were highest at the sites of inflammation. On the other hand, treatment with dexamethasone, an immunosuppressive drug, abrogated EMT and cancer invasiveness. These results imply that inflammation can be a cancer progression factor by facilitating the EMT process in cancer cells^54^. Similarly, localized inflammation in the lungs can trigger cancer cell escape from dormancy, which leads to the development of macroscopic metastases^30^. During this process, Zeb1 expression, a strong inducer of EMT, was required for cancer cells to escape dormancy. On the other hand, depletion of neutrophils via the administration of antibodies against the lymphocyte antigen 6 complex, locus G (Ly6G) abrogated the reactivation of DCCs.

The interaction between cancer cells and myeloid cells has also been implicated in cancer progression. For instance, inflammatory monocytes with Ly6C expression can facilitate the extravasation of cancer cells in the lung by secreting chemokine C-C-motif ligand 2 (CCL2)^55^ and vascular endothelial growth factor^56^. Then, macrophages bind to cancer cells and increase the survival of cancer cells. In this procedure, vascular cell adhesion protein 1 on cancer cells binds to β-1-integrin-positive macrophages, and this interaction activates Akt signaling in cancer cells and allows them to evade TNF-related apoptosis-inducing ligand (TRAIL)-induced apoptosis^57^. Together, these mechanistic roles of myeloid cells are related to metastasis-promoting effects. However, whether the interaction between myeloid cells and cancer cells switches on the growth of DCCs has not yet been sufficiently demonstrated.

The differentiation of monocytes into metastasis-associated macrophages (MAMs) can promote the metastatic outgrowth of cancer cells. The metastasis-promoting role of MAMs is more complicated and related to their participation in sculpting a more fibrotic metastatic microenvironment. In a genetic mouse model of pancreatic ductal adenocarcinoma (PDAC), MAMs secreted granulin in the liver, and granulin induced the transformation of resident hepatic stellate cells into myofibroblasts. In turn, myofibroblasts secreted periostin, which created a fibrotic microenvironment that was more favorable for integrin singling activation^58^. Then, activated integrin signaling led to DCC reactivation and promoted the proliferation of cancer cells at the metastatic lesion. Therefore, the development of a more fibrotic metastatic microenvironment by MAMs can function as a prometastatic factor by awakening DCCs.

The involvement of natural killer (NK) cells in cancer dormancy and reactivation has not yet been determined, and instead, it has been elucidated that DCCs are more resistant to the cytotoxicity of NK cells. In a “latency-competent cancer model” where dormant clones were selected from an in vivo experimental metastasis assay, DCCs were confirmed to activate the p38 and self-renewal pathways through Sox2/9. Sox2 was also shown to facilitate DKK1 secretion and thereby inhibit Wnt signaling as well as downstream proliferative pathways^35^. Once DCCs enter dormancy via DKK1, they are able to avoid NK cell-mediated cell death, while DCCs with low DKK1 expression are still proliferative and susceptible to NK cell cytotoxicity.

Recently, a growing body of evidence has highlighted the potential role of CD4 and CD8 T cells in cancer dormancy maintenance^59,60^. DCCs were far less susceptible to adaptive immunity and showed low expression of cancer antigen. Additionally, dormant leukemia cells were confirmed to express PDL-1, which allows them to avoid T cell-mediated cytotoxicity^59,60^. These findings indicate that DCCs can escape anticancer immunity, thereby surviving for an extended period. Additionally, an in vivo xenograft model using dormant murine breast cancer cell clones selected with constitutive treatment of doxorubicin has revealed that both CD8 and CD4 T cells are involved in chemotherapy-mediated dormancy as well^61^. Chemotherapy treatment activated IFN signaling in cancer cells through an autocrine and self-sustained increase in TF and interferon regulatory factor 7 (IRF7). IRF7/IFN signaling promoted the expansion of CD4 and CD8 T cells and prevented the mobilization of CD11b^+^Gr1^+^ myeloid-derived suppressor cells. Collectively, these effects facilitate immune cytotoxicity, resulting in immune-mediated cancer dormancy.

More recently, neutrophils have attracted significant attention because of their DCC-reawakening activity. Exposure to tobacco smoke or the nasal instillation of lipopolysaccharide induced chronic lung inflammation and converted DCCs to aggressively growing cancer cells, resulting in an increase in metastasis. In this process, neutrophils mediated the DCC reawakening through the formation of neutrophil extracellular traps (NETs), which are scaffolds of chromatin, including cytotoxic enzymes and proteases that are released into the extracellular space^62^. Mechanistically, two proteases, neutrophil elastase and matrix metalloproteinase 9 (MMP9), were secreted from NETs and sequentially cleaved and remodeled laminin. In turn, the remodeled laminin activated integrin α3β1 signaling in DCCs and promoted their proliferation. Treatment with antibodies against NET-remodeled laminin prevented the awakening of DCCs and reduced metastasis.

## Summary and direction of future research

In inhospitable microenvironments, cancer cells may enter a state of dormancy to protect themselves against apoptotic and antiproliferative treatments so that the fittest may survive^63,64^. The existence of DCCs has led to the emergence of therapy resistance, and most importantly, the cells may resume growth, raising the risk of lethal metastatic outbreaks even after a long latency period of months to years. For these reasons, DCCs have been attracting significant interest as a therapeutic target for improving clinical outcomes. The removal of DCCs in combination with antiproliferative treatment is one therapeutic option; however, cellular and surface markers for DCCs are mostly unavailable at present. An overwhelming number of reports propose that DCC reawakening is the final step of the metastatic outbreak, so blocking the factors responsible for this process is key to preventing poor clinical outcomes. Although a variety of signaling cascades are linked to the breaking of dormancy, these signaling networks eventually lead to a change in the balance between p38 and ERK activities in favor of ERK^8^. Therefore, if we can finely modulate the balance of p38 and ERK, we may be able to induce permanent dormancy and prevent metastasis, which will mark a new era of cancer treatment.

**Fig. 1:**
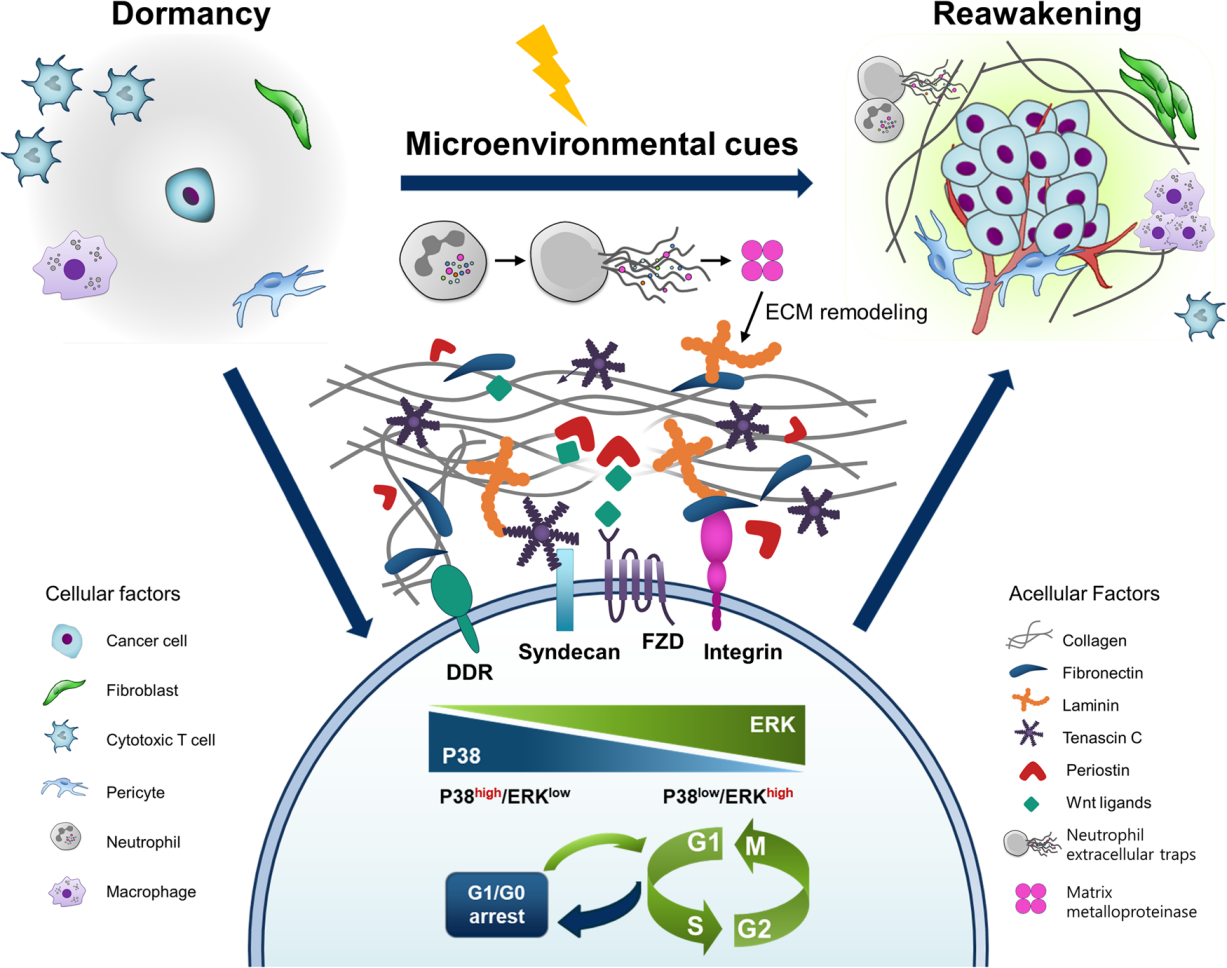
Schematic view of cellular and acellular factors that reawaken dormant cancer cells. Cancer cells often enter dormancy to evade immune attack. Once in a new location, these dormant cancer cells (DCCs) receive signals from the surrounding tissue, thereby gain the ability to re-enter the cell cycle. Also, chronic inflammation can reactivate DCCs, which can trigger tumor development. Key signaling components involved in DCC reactivation are currently being investigated and may help to fight this leading cause of death from cancer. ECM, extracellular matrix; DDR, discoidin domain receptor; FZD, frizzled.

In the present review, we provide an overview of the cellular and acellular mechanisms that break the dormancy-permissive p38^high^/ERK^low^ status (Fig. 1). During their journey in the blood and lymphatic stream, DCCs do not interact with local cells or the ECM. However, once they reach an organ, they encounter a new combination of ECM, growth factors, and cytokines produced from local stromal and immune cells. The binding of fibronectin to integrins has a fundamental role in shifting the balance of p38 and ERK activities in favor of ERK. Additionally, other ECM components, such as tenascin C and periostin secreted from resident stromal cells, can foster the binding of fibronectin and integrins and can therefore act as substantial DCC-reawakening factors. In addition, chronic inflammation can initiate the regrowth of DCCs through integrin activation. Macrophages promote the secretion of fibronectin from nearby fibroblasts and sculpt a more fibrotic metastatic microenvironment, thereby fostering the binding of fibronectin to integrin on DCCs. Additionally, neutrophils participate in ECM remodeling by secreting proteinase enzymes, sequentially activating integrin signaling, and reawakening DCCs. Other immune cells, such as monocytes and myeloid cells, have functional involvement in triggering escape from dormancy in multiple experimental models, although their necessity in integrin signaling activation has not yet been tested. Several target molecules that are involved in DCC reawakening are currently under clinical investigation for cancer therapy or prevention as single or combinatory agents (Table 1). Although some of the trials have been terminated because of limited efficacy and intolerable side effects, some have shown promising clinical results, such as a significant trend toward improved disease-free survival and tumor reduction with minimal side effects. Therefore, further investigation into the microenvironmental cues that favor integrin and p38^low^/ERK^high^ activity would broaden the current knowledge of DCC-reawakening factors.

**Table 1:** Potential molecular targets and therapeutic agents linked to the DCC reawakening mechanism.

Target	Drug	Strategy	Clinical trial	Treatment	Current status	Disease	Clinical results
Integrin β1	ATN-161	Non-RGD-based integrin-binding peptide	Phase I/II (NCT00352313)	Combinatory (with carboplatin)	Completed	Recurrent malignant glioma	All of the treatment-related adverse events were grade 2 or lower^65^.
			Phase II (NCT00131651)	Single	Terminated	Advanced renal cell carcinoma	Unposted
Integrin α5β1	Volociximab	Chimeric monoclonal antibody against integrin α5β1	Phase Ib (NCT00666692, NCT00654758)	Combinatory (with carboplatin, paclitaxel, bevacizumab)	Completed	Advanced non-small-cell lung cancer (NSCLC)	Treatment was well tolerated, and dose-limiting toxicities were not observed. Approximately one-quarter of patients displayed stable disease^66^.
			Phase II (NCT00369395)	Single	Terminated	Metastatic melanoma	Terminated because of insufficient clinical activity
			Phase II (NCT00099970)	Combinatory (with dacarbazine)	Completed	Metastatic melanoma	Unposted
			Phase II (NCT00516841)	Single	Terminated	Platinum-resistant advanced epithelial ovarian cancer, primary peritoneal cancer	Terminated based on lack of efficacy^67^
			Phase II (NCT00401570)	Combinatory (with gemcitabine)	Completed	Metastatic pancreatic cancer	Unposted
			Phase II (NCT00100685)	Single	Terminated	Metastatic renal cell carcinoma	Unposted
			Phase I/II (NCT00635193)	Combinatory (with doxorubicin)	Completed	Ovarian cancer, primary peritoneal cancer	Unposted
Integrin α2	E7820	Oral inhibitor of integrin alpha-2 expression (sulfonamide-based small molecule)	Phase I (NCT01773421)	Single	Completed	Advanced solid tumors	E7820 decreases integrin alpha-2 in surrogate tissues and is associated with stable disease^68^.
			Phase I/II (NCT01347645)	Single	Completed	Locally advanced/metastatic colon/rectal cancer	E7820 treatment was safe and tolerable in 2/3 of patients^69^.
			Phase I/II (NCT01133990)	Combinatory (with FOLIRI)	Completed	Locally advanced/metastatic colon/rectal cancer	Limited efficacy in locally advanced or metastatic colorectal carcinoma^69^.
			Phase II (NCT00309179)	Combinatory (with cetuximab)	Completed	Advanced colorectal cancer	E7820 combined with cetuximab is well tolerated. A single partial response was observed in a total of seven KRAS-mutant pateints^70^.
			Phase I (NCT00078637)	Single	Completed	Neoplasms, lymphoma, malignant cancers	Unposted
Integrin αv	Intetumumab (CNTO-95)	Panintegrin αv antibody	Phase I (NCT00888043)	Combinatory (with Avastin)	Completed	Solid tumors	Unposted
			Phase II (NCT00537381)	Combinatory (with docetaxel and prednisone)	Completed	Metastatic hormone-refractory prostate cancer	Treatment resulted in shorter progression-free survival without additional toxicity^71^.
			Phase I/II (NCT00246012)	Single or combinatory (with dacarbazine)	Completed	Melanoma (stage 4)	CNTO-95 showed a favorable safety profile and nonsignificant effects on overall survival^72^.
Integrin αv	Abituzumab	Panintegrin αv antibody	Phase I (NCT00848510)	Single	Completed	Colorectal/ovarian cancer with liver metastases	It was tolerable despite hypersensitivity reactions^73^.
			Phase I/II (NCT01008475)	Combinatory (with irinotecan and cetuximab)	Completed	Kras-wild-type metastatic colorectal cancer	A trend toward improved overall survival was observed^74^.
			Phase II (NCT01360840)	Single	Completed	Asymptomatic/mildly symptomatic metastatic castrate-resistant prostate cancer	Although progression-free survival was not significantly extended, abituzumab appears to have specific activity in prostate cancer-associated bone lesions^75^.
Integrin αvβ3	Etaratuzumab (MEDI-522)	Humanized higher-affinity variants derived from murine antibody LM609	Phase I/II (NCT00027729)	Single	Completed	Advanced colorectal cancer	Unposted
			Phase I (NCT00049712)	Single	Completed	Refractory advanced solid tumors, lymphoma	Unposted
			Phase I/II (NCT00284817)	Single	Completed	Irinotecan-refractory advanced colorectal cancer	Unposted
			Phase II (NCT00072930)	Combinatory (with docetaxel, prednisone, zoledronic acid)	Completed	Metastatic androgen-independent prostate cancer	Unposted
			Phase I/II (NCT00684996)	Combinatory (with bevacizumab)	Terminated	Unresectable/metastatic kidney cancer	Posted
			Phase I (NCT00263783)	Single	Completed	Refractory solid tumors	Unposted
			Phase II (NCT00066196)	Single or combinatory (with dacarbazine)	Completed	Metastatic melanoma	MEDI-522 appears to be well tolerated. The overall survival results suggested potential clinical activity of MEDI-522^76^.
			Phase I (NCT00111696)	Single	Completed	Advanced malignant melanoma	Unposted
Integrin αv	MK-0429	An equipotent inhibitor of multiple αv integrins	Phase I (NCT00302471)	Single	Completed	Prostate cancer with metastatic bone disease	MK‐0429 was generally well tolerated and a reduction in bone turnover was observed^77^.
Integrin αvβ3 and αvβ5	Cilengitide (EMD121974)	A constrained cyclic pentapeptide based on the RGD sequence	Phase II (NCT00103337)	Single	Completed	Metastatic prostate cancer	Unposted
			Phase II (NCT00089388)	Single	Terminated (administratively complete)	Acute myeloid leukemia	Unposted
			Phase I (NCT00063973)	Single	Completed	Children with refractory primary brain tumors	Unposted
			Phase I (NCT01118676)	Combinatory (with radiochemotherapy)	Completed	Locally advanced NSCLC	Unposted
			Phase II (NCT00679354)	Single	Completed	Recurrent/Progressive high-grade glioma that has not responded to a standard regimen	Posted
			Phase I (NCT00022113)	Single	Completed	Advanced solid tumors	Dose-limiting toxicity was not observed^78^.
			Phase II (NCT00121238)	Single	Completed	Prostate cancer	Cilengitide was well tolerated but had no detectable clinical activity^78^.
			Phase II (NCT00093964)	Single	Completed	Recurrent glioblastoma multiforme	Posted
			Phase II (NCT01517776)	Combinatory (with temozolomide)	Terminated (due to an altered benefit/risk ratio)	Refractory high-grade gliomas, diffuse intrinsic pontine gliomas in children and adolescents	Unposted
			Phase I (NCT00077155)	Single	Completed	Advanced solid tumors, lymphoma	Unposted
			Phase I/II (NCT00006093)	Single	Completed	Progressive/recurrent glioma	No dose-limiting toxicity was observed^78^.
uPA	WX-671	Orally available prodrug of WX-UK1	Phase II (NCT00499265)	Combinatory (with gemcitabine)	Completed	Locally advanced pancreatic cancer that cannot be removed by surgery	More patients achieved a partial response with WX-671 combination therapy than with standard of care^79^.
			Phase II (NCT00615940)	Combinatory (with Capecitabine)	Completed	Her2-negative metastatic breast cancer	Unposted
	WX-UK1	A serine protease inhibitor that inhibits uPA as well as other serine proteases	Phase I (NCT00083525)	Combinatory (with capecitabine)	Completed	Advanced malignancies	Unposted
FAK	GSK2256098	A tyrosine kinase inhibitor working at the autophosphorylation site (Tyr 397) of FAK	Phase I (NCT01938443)	Combinatory (with trametinib)	Completed	Advanced solid tumors	Trametinib exposure was increased in combination with GSK2256098. Clinical efficacy was limited in combinatory therapy. The safety profile was acceptable^80^.
			Phase I (NCT01138033)	Single	Completed	Solid tumors	GSK2256098 has an acceptable safety profile and has clinical activity in patients with mesothelioma, particularly those with merlin loss^81^.
			Phase I (NCT00996671)	Single	Completed	Healthy volunteers	Unposted
			Phase II (NCT02523014)	Single	Suspended (not currently open to patient registration)	Intracranial meningioma, recurrent meningioma with NF2 gene mutation	Unposted
			Phase II (NCT02428270)	Combinatory (with trametinib)	Active, not recruiting	Advanced pancreatic cancer	The GSK2256098 and trametinib combination was well tolerated but was not effective in patients^82^.
	VS-4718	VS-4718 blocks fibronectin-stimulated FAK autophosphorylation at Tyr397	Phase I (NCT02651727)	Combinatory (with paclitaxel and gemcitabine)	Terminated	Pancreatic cancer	Unposted
			Phase I (NCT01849744)	Single	Terminated (sponsor’s decision to deprioritize the program)	Nonhematologic cancers, metastatic cancer	Unposted
			Phase I (NCT02215629)	Single	Withdrawn	Acute myeloid leukemia, B cell acute lymphoblastic leukemia	–
	VS-6063 (defatinib)	VS-6063 inhibits FAK phosphorylation at the Tyr397	Phase I (NCT00787033)	Single	Completed	Advanced nonhematologic malignancies	VS-6063 has an acceptable safety profile. Treatment-related adverse events were mild to moderate, and reversible^83^.
			Phase I (NCT01943292)	Single	Completed	Nonhematologic cancers	Posted
			Phase I/Ib (NCT01778803)	Combinatory (with paclitaxel)	Completed	Advanced ovarian cancer	Defactinib was generally well tolerated in combination with weekly paclitaxel^84^.
			Phase I (NCT03875820)	Combinatory (with RO5126766)	Recruiting	NSCLC, solid tumors, low-grade serous ovarian cancer, colorectal cancer	–
			Phase I (NCT02546531)	Combinatory (with pembrolizumab and gemcitabine)	Active, not recruiting	Advanced solid tumors, solid tumors, pancreatic cancer	–
			Phase I/II (NCT02758587)	Combinatory (with pembrolizumab)	Recruiting	Carcinoma, NSCLC, mesothelioma, pancreatic neoplasm	–
			Phase II (NCT01951690)	Single	Completed	Non-small-cell lung cancer, lung cancer	Defactinib monotherapy showed modest clinical activity in heavily pretreated patients with KRAS mutation^85^.
			Phase II (NCT02004028)	Single	Terminated (company decided to discontinue trial to focus on development program next steps)	Surgically resectable malignant pleural mesothelioma	Unposted
			Phase II (NCT03727880)	Combinatory (with pembrolizumab)	Recruiting	Resectable pancreatic ductal adenocarcinoma	–
PKC	LXS196	Small-molecule inhibitor for PKC	Phase I (NCT02601378)	Single or combinatory (with HDM201)	Active, not recruiting	Metastatic uveal melanoma	–
JAK2	Pacritinib (SB1518)	Macrocyclic pyrimidine-based JAK2 inhibitor	Phase I (NCT02342353)	Combinatory (with erlotinib)	Terminated (drug shortage)	EGFR-mutant NSCLC	Unposted
			Phase I (NCT02323607)	Combinatory (with chemotherapy)	Completed	Acute myeloid leukemia, FLT3 mutations	Unposted
			Phase I (NCT03601819)	Single	Recruiting	Relapsed/refractory lymphoproliferative disorders	–
			Phase II (NCT02277093)	Single	Terminated (FDA issued a clinical hold as pacritinib had increased side effects)	Refractory colorectal cancer	Posted
			Phase I/II (NCT00719836)	Single	Completed	Advanced myeloid malignancies	Pacritinib showed clinical activity in myelofibrosis with tolerable side effects^86^.
			Phase II (NCT02532010)	Combinatory (with decitabine or cytarabine)	Terminated (initially by the sponsor and later due to financial constraints)	Older patients with acute myeloid leukemia	Posted
	Ruxolitinib	Small-molecule inhibitor of JAK1/2	Phase II (NCT01877005)	Single	Completed	Hodgkin’s lymphoma	Unposted
			Phase II (NCT02876302)	Combinatory (with preoperative chemotherapy)	Recruiting	Triple-negative inflammatory breast cancer	–
			Phase II (NCT01423604)	Combinatory (with capecitabine)	Completed	Pancreatic cancer	Treatment was generally well tolerated and may have improved survival in patients with metastatic pancreatic cancer with evidence of systemic inflammation^87^.
			Phase II (NCT01594216)	Combinatory (with exemestane)	Completed	Estrogen receptor-positive breast cancer	Unposted
			Phase I/II (NCT02066532)	Combinatory (with trastuzumab)	Active, not recruiting	Metastatic HER2-positive breast cancer	–
			Phase I/II (NCT02041429)	Combinatory (with preoperative chemotherapy)	Active, not recruiting	Triple-negative inflammatory breast cancer	–
			Phase II (NCT03153982)	Single	Recruiting	Operable head and neck cancer	–
			Phase II (NCT00674479)	Single	Completed	Advanced hematologic malignancies	Posted
			Phase I/II (NCT02155465)	Combinatory (with erlotinib)	Completed	EGFR-mutant lung adenocarcinoma with acquired resistance to erlotinib	Posted
			Phase I (NCT01702064)	Combinatory (with nilotinib)	Completed	Chronic myeloid leukemia	The combinatory treatment was safe and tolerable, and the molecular responses were encouraging^88^.
			Phase I/II (NCT01751425)	Combinatory (with tyrosine kinase inhibitors)	Active, not recruiting	Chronic myeloid leukemia with minimal residual disease while on therapy with tyrosine kinase inhibitors	The combinatory treatment was safe and tolerable. There was no apparent clinical benefit^89^.
	AZD1480	ATP-competitive inhibitor of JAK1 and 2 kinases	Phase I (NCT01219543)	Single	Terminated (compound development discontinued)	Solid tumors, advanced solid malignancies, advanced hepatocellular carcinoma, EGFR- and/or ROS-mutant non-small-cell lung cancer, lung carcinoma metastasis, gastric cancer	Unposted
			Phase I (NCT01112397)	Single	Terminated (decision to stop development of AZD1480)	Solid tumors	Unposted
STAT3	WP1066	Dephosphorylation and nuclear export of constitutively phosphorylated STAT3	Phase I (NCT01904123)	Single	Recruiting	Recurrent malignant glioma, progressive metastatic melanoma in the brain	–
	AZD9150	STAT3 antisense oligonucleotide	Phase I (NCT03527147)	Combinatory (with acalabrutinib)	Recruiting	Relapsed/refractory aggressive non-Hodgkin’s lymphoma	–
			Phase I/II (NCT03421353)	Combinatory (with durvalumab or chemotherapy)	Active, not recruiting	Advanced solid tumors	–
			Phase I/Ib (NCT01839604)	Single	Completed	Advanced/metastatic hepatocellular carcinoma	Posted
			Phase I/II (NCT01563302)	Single	Completed	Advanced cancers	AZD9150 was well tolerated and showed efficacy in a subset of heavily pretreated patients with diffuse large B cell lymphoma^90^.
	OPB-51602	A small-molecule SH2 domain-targeting STAT3 inhibitor	Phase I (NCT02058017)	Single	Terminated (because of unbearable lactic and metabolic acidosis)	Locally advanced nasopharyngeal carcinoma	Unposted
			Phase I (NCT01423903)	Single	Completed	Advanced cancer	Unposted
			Phase I (NCT01344876)	Single	Completed	Hematologic malignancies	OPB-51602 was safe and well tolerated. However, long-term administration at higher doses was difficult with the daily dosing schedule, and no response was seen^91^.
			Phase I (NCT01184807)	Single	Completed	Advanced solid tumors	OPB-51602 demonstrated promising antitumor activity, particularly in NSCLC. Less frequent dosing should be explored^92^.
COX1/2	Sulindac	Nonsteroidal anti-inflammatory drug (NSAID), arylalkanoic acid derivative	Phase III (NCT00118365)	Combinatory (with eflornithine)	Completed	Preventing colorectal cancer with colon polyps	Posted
			Phase III (NCT01349881)	Single and combinatory (with eflornithine)	Recruiting	Reducing the three-year event rate of adenomas and second primary colorectal cancers in patients previously treated for stages 0 through III colon/rectal cancer	–
			Phase I (NCT00245024)	Single	Completed	Preventing breast cancer in women at high risk of breast cancer	Unposted
			Phase II (NCT01856322)	Single	Terminated (due to lack of accrual)	Advanced colorectal cancer	–
			Phase II (NCT00039520)	Combinatory (with docetaxel)	Completed	Metastatic/recurrent breast cancer	Unposted
			Phase II (NCT00368927)	Single	Completed	Preventing lung cancer in current/former smokers with bronchial dysplasia	Sufficient benefits were not observed^93^.
	Celecoxib	a COX-2-selective NSAID	Phase II (NCT01695226.)	Single	Completed	Preoperative celecoxib treatment in breast cancer	Celecoxib induced transcriptional programs supporting antitumor activity^94^.
			Phase III (NCT 01041781)	Combinatory (with gemcitabine, pemetrexed disodium and carboplatin)	Terminated (recommended by the Data and Safety Monitoring Board)	Advanced NSCLC	A urinary metabolite of prostaglandin E2 was able to identify patients who could benefit from COX2 inhibition^95^.
			Phase III (NCT 02429427)	Single	Completed	Primary breast cancer	Clinical benefit was not observed. Further studies focusing on the ER + subpopulation are ongoing^96,97^.
